# Can Gut Microbiota Analysis Reveal *Clostridioides difficile* Infection? Evidence from an Italian Cohort at Disease Onset

**DOI:** 10.3390/microorganisms13010016

**Published:** 2024-12-25

**Authors:** Roberto Rosato, Gianluca Quaranta, Giulia Santarelli, Giovanni Fancello, Delia Mercedes Bianco, Francesca Romana Monzo, Stefano Bibbò, Giovanni Cammarota, Maurizio Sanguinetti, Luca Masucci, Flavio De Maio

**Affiliations:** 1 Department of Basic Biotechnological Sciences, Intensivology and Perioperative Clinics, Section of Microbiology, Università Cattolica del Sacro Cuore, 00168 Rome, Italy; 2 Department of Laboratory and Infectious Sciences, Fondazione Policlinico Universitario “A. Gemelli” IRCCS, 00168 Rome, Italy; 3 Hull University Teaching Hospitals NHS Trust, Hull HU3 2JZ, UK; 4 Department of Translational Medicine and Surgery, Università Cattolica del Sacro Cuore, 00168 Rome, Italy; 5 Department of Medical and Surgical Sciences, UOC Gastroenterologia, Fondazione Policlinico Universitario Agostino Gemelli IRCCS, 00168 Rome, Italy; 6 Department of Medical and Surgical Sciences, UOC CEMAD Centro Malattie dell’Apparato Digerente, Medicina Interna e Gastroenterologia, Fondazione Policlinico Universitario Agostino Gemelli IRCCS, 00168 Rome, Italy

**Keywords:** *Clostridioides difficile*, CDI infection, gut microbiota

## Abstract

A diverse and well-functioning gut microbiota normally serves as a protective shield against the invasion of harmful bacteria or the proliferation of opportunistic pathogens. *Clostridioides difficile* infection (CDI) is predominantly associated with the overuse of antibiotics, resulting in a significant alteration in the gut’s microbial balance. Unfortunately, the lack of global standardization does not allow for the identification of a set of biomarkers associated with the onset and progression of this disease. In this study, we examined the composition of the gut microbiota in patients at the time of the initial detection of CDI compared to a control group of CDI-negative individuals, with a focus on identifying potential CDI biomarkers for diagnosis. While no significant differences in the alpha and beta diversity between CDI-negative and CDI-positive individuals were found, we found that certain genera (such as *Clostridium* XIVa and *Clostridium* XVIII) showed different abundance patterns in the two groups, indicating potential differences in gut microbiota balance. In conclusion, am enrichment in *Clostridium* XI and a decrease in *Faecalibacterium* emerged in the CDI-positive patients and following antibiotic treatment, indicating that changes in the *Clostridium/Faecalibacterium* ratio may be a promising biomarker that warrants further investigation for CDI diagnosis.

## 1. Introduction

*Clostridioides difficile* (*C. difficile*) is a spore-forming Gram-positive bacterium belonging to the *Firmicutes* phylum which can cause a highly transmissible infection (CDI) that occurs mainly in hospitalization facilities [1]. Its symptoms can range from asymptomatic colonization to fearsome complications such as toxic megacolon [2,3]. The onset of CDI is commonly associated with nonspecific signs and symptoms including diarrhea, fever, abdominal pain, and an increased white blood cell count [4]. Laboratory detection of the Glutamate Dehydrogenase (GDH) antigen and toxins A/B confirms a diagnosis [5,6,7]. CDI appears to primarily be linked to the excessive use of antibiotics, which causes significant perturbation of the gut’s microbial community [8,9,10]. Indeed, a healthy gut microbiota provides a protective environment against the colonization of pathogenic bacteria or the overgrowth of opportunistic pathogens, including *C. difficile* [11,12].

In this scenario, various studies have described significant alterations in the gut microbiota of adults with CDI without clarifying these interactions definitely [13]. In particular, a significant reduction in the abundance of *Firmicutes* has been documented in instances of CDI [6,7]. This decrease is mainly attributed to important families such as *Lachnospiraceae* and *Ruminococcaceae*, which play a fundamental role in promoting gut health by producing short-chain fatty acids (SCFAs), including butyrate, known for its anti-inflammatory effects and its support of intestinal barrier function [14,15]. Conversely, the opposite observation has been reported for the *Bacteroidetes* phylum: whereas some studies have indicated a general decrease in its abundance [16,17,18], others have demonstrated a significant increase [19,20].

Additionally, genera such as *Blautia*, *Roseburia*, *Anaerostipes*, *Faecalibacterium*, *Collinsella*, and *Coprococcus* are notably diminished in individuals with CDI. Conversely, *Enterococcus*, *Veillonella*, and *Lactobacillus* spp. display increased abundance in these patients [7,8,9]. These modifications in terms of the abundance of certain bacteria may contribute to the dysbiosis observed in CDI, potentially favoring the growth of pathogenic bacteria and dysregulating the host’s immune responses [21,22].

Investigations into the gut microbiota of patients with asymptomatic *C. difficile* colonization indicate that their microbial composition is more closely aligned with that of healthy adults. The most prevalent phyla remain *Bacteroidetes*, *Firmicutes*, and *Proteobacteria*, although specific alterations have been noted [10]. On the other hand, a reduction in the families *Bacteroidaceae* and *Prevotellaceae* has been observed within *Bacteroidetes*. On the other side, a reduction in the genera *Prevotella*, *Alistipes*, and *Bacteroides* has been reported, followed by an observed increase in *Parabacteroides*, *Peptostreptococcaceae*, and *Enterococcus* [7,17,20,23]. These findings suggest that while asymptomatic carriers maintain a microbial profile closer to that of healthy individuals, subtle shifts in the abundance of key taxa may occur, possibly influencing the transition from colonization to infection.

The current diagnostic tests for CDI fail to assess *C. difficile* levels within the broader context of a patient’s microbial communities, making the identification of specific microbial signatures associated with susceptibility or resistance to CDI virtually impossible. Understanding the dynamics of the microbial community during the onset of CDI could provide valuable insights into the interplay between pathogens and commensal bacteria, contributing to the development of targeted therapeutic interventions [2,13,24].

Additionally, the use of 16S rRNA gene sequencing for microbiome profiling could improve preventive measures tailored to individuals with a heightened risk, ultimately enhancing the effectiveness of CDI management strategies [25,26].

In other words, further research is required to elucidate the complex interactions between microbial composition (and host immunity) and CDI progression. Unfortunately, neither a unique sequencing approach nor standardized bioinformatics pipelines are yet available for 16s metabarcoding [27,28,29]. In this scenario, we analyzed the gut microbiota at the first diagnosis of CDI and the onset of showing symptoms, comparing the results obtained by sequencing different 16s RNA hypervariable regions to assess the accuracy in the identification of *C. difficile* and related species which may be relevant biomarkers for monitoring of CDI-related microbiota.

## 2. Materials and Methods

### 2.1. Study Population and CDI Detection

Adult patients (age > 18 years old) hospitalized at the Fondazione Policlinico Universitario Agostino Gemelli IRCCS who showed gastrointestinal symptoms and were suspected of CDI or required microbiota investigation were considered eligible for this study. One hundred and twenty patients were recruited and equally stratified into two groups based on *C. difficile* Glutamate Dehydrogenase (GDH) and toxin A/B (TOX A/B) detection using a Chemiluminescence Immunoassay (CLIA) (the Liaison system—DiaSorin Spa, Saluggia VC, Italy). According to the international guidelines, the laboratory examination was carried out only on unformed diarrheal stools, and a positive test was defined as the detection of both targets [30]. Patients previously diagnosed with CDI, with recurrent CDI, and positive for GDH but negative for TOXs A/B were excluded.

### 2.2. Sample Manipulation, DNA Extraction, and Library Preparation

Fresh stool samples were collected when a microbiological diagnosis of CDI was made and were stored at −80 °C until they were processed. The DNA extraction was performed in a strictly controlled, level 2 biological safety workplace. In keeping with the previously described protocol, we suspended a 200 mg fecal sample in hexadecyltrimethylammonium bromide (CTAB) buffer, and this suspension was used to extract bacterial DNA using the DANAGENE MICROBIOME Fecal DNA kit (Danagen-Bioted, Barcelona, Spain) [31]. The DNA was eluted in 200 µL of pre-heated nuclease-free water and stored at −20 °C until it was processed [32]. Subsequently, DNA quantification was performed using a Qubit 4.0 fluorometer (Life Technologies, Carlsbad, CA, USA), and the extracted DNA was then stored at −20 °C.

The V1-V3 and V3-V4-V6 hypervariable regions of the 16S rRNA gene were amplified by using Microbiota solution A (sol.A) and solution B (sol.B) (Arrow Diagnostics), respectively [33]. The extracted DNA (5.0 ng) was used as the template in a 20 µL PCR volume, which contained Enzyme Mix 1 and Amp Mix (sol.A or sol.B) solutions. The thermal cycling conditions were set as follows: (i) 95 °C for 5 min; (ii) 25 cycles, each consisting of 95 °C for 30 s, 55 °C for 30 s, and 72 °C for 30 s; and (iii) 72 °C for 5 min. The amplicons were purified using 29.5 µL Agencourt AMPure XP beads (Beckman Coulter, Mumbai, Maharashtra), which had previously been diluted in 19.0 µL of nuclease-free water, and were eluted in 35 µL of nuclease-free water. For each sample, the Enzyme Mix 2 (10 µL) solution and nuclease-free water (8 µL) were mixed, and the final suspension was used to harvest the assigned lyophilized index. To each recovered index, 2 μL of the previous amplicon was added. The thermal cycling conditions were set as follows: (i) 95 °C for 5 min; (ii) 8 cycles, with each cycle consisting of 95 °C for 30 s, 65 °C for 30 s, and 72 °C for 30 s; and (iii) 72 °C for 5 min. The indexed amplicons were purified using Agencourt AMPure XP beads, as described above, and were eluted in 25 µL of 10 mM Tris/1 mM EDTA, at a pH of 8.0. The amplicons were then checked for their quality on 1% agarose gel electrophoresis (Thermo Fisher, Waltham, MA, USA), and the DNA concentration was determined using the above-mentioned method. The final amplicon length was 640 bp and 900 bp for sol.A and sol.B, respectively.

Each sample’s indexed amplicons were equimolarly diluted, and the final pool (5 pM) was subjected to 2 × 250 paired-end sequencing (Illumina) using an Illumina MiSeq instrument [32]. To enhance the base diversity, the internal control PhiX v3 (Illumina, San Diego, CA, USA) was introduced into the final library.

### 2.3. Bioinformatics Analyses

The raw data were first analyzed by using MicrobAT (Microbiota Analysis Tool) v. 1.1.0 suite software provided by SmartSeq bioinformatics. Briefly, the raw paired-end reads, exported as a FASTQ.gz file, were cleaned up to remove sequences with a short read length (i.e., <200 nucleotides) and a low quality (i.e., with an average Phred quality score of <25). Taxonomic assignment of the sequences was performed by aligning all of the sequences derived from the previous selection process described above with the Ribosomal Database Project (RDP) reference database (v. 11.5). Sequences that met the criteria of a minimum sequence length alignment with the RDP database of ≥80% and a similarity threshold of ≥97% were clustered to the taxonomic level of species [34]. A biological observation matrix (BIOM) was generated at different taxonomic levels.

The downstream data analysis was performed using R (v. 4.0.2, https://www.rstudio.com/) and the phyloseq package (v1.46) [35] with an in-house analysis pipeline. Firstly, unclassified bacteria and archaeal ASVs (each represented by a single ASV) were removed. Data filtering was performed to remove features that were unlikely to contribute meaningfully to the data modeling. Features with a low count and variance were removed during the filtration step (ASVs detected less than 3 times and not in at least 10% of the samples), while those with very few counts were filtered based on their median abundance levels across samples (*Candidatus Saccharibacteria*, *Lentisphaerae*, and *Synergistetes*). To minimize the effect of differences in the sequencing depth, for alpha diversity and for the ordination analysis (beta diversity), samples were normalized using the median sequencing depth.

To measure the alpha diversity, we used the final dataset to calculate the Shannon index and Pielou’s evenness. Statistical significance was assessed by using the Wilcoxon signed rank test. To measure the beta diversity, we used the final dataset to generate a Bray–Curtis distance matrix (at the taxon level), which was then represented using a principal coordinate analysis (PCoA). Statistical significance was assessed using the vegan package’s (v2.6-4) adonis function, which performs a permutational multivariate analysis of variance (PERMANOVA). Relative abundance was finally computed at the phylum and genus levels, and statistical significance was assessed using the Wilcoxon signed rank test [36].

## 3. Results

### 3.1. Demographic and Clinical Profiles

**Figure 1:**
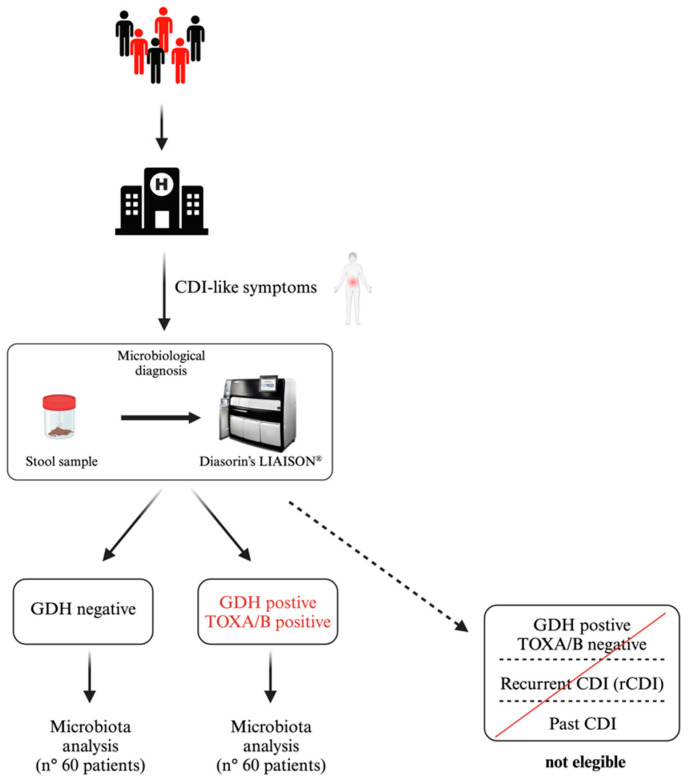
Representative scheme of the criteria used for the selection of patients within the CDI-positive and CDI-negative groups.

A total of 120 patients investigated for CDI were gathered into two groups: *C. difficile*-infection-positive, as defined by a positive GDH screening and being positive for TOXs A/B (n. 60 patients), and a *C. difficile*-infection-negative cohort, defined by a negative GDH screening (n. 60 patients) (Figure 1). Our cohort was mostly homogenous in terms of age, gender, and clinical signs (unformed diarrheal stools were common to all patients). Importantly, of the patients with a positive test showing the first detection of CDI (not associated with severe disease), 38.3% had community-acquired CDIs [37].

The CDI-positive group showed a median age of 75.50 years [62.00–82.00] and a median hospital stay of 9.50 [2.00–24.75] days. Among the CDI-positive patients, 26 (43.3%) were female. Conversely, the CDI-negative individuals had a median age of 62 [48.50–76.00] and a minor length of hospital stay, which accounted for a median of 4.50 [1.00–10.75] (*p* > 0.05) (see Table 1 for details).

In this group, the GDH values were above the maximum limit of detection (>95 IU/mL) in all patients, while their TOX A/B values appeared to be more variable, with a median value of 10.15 [3.17–71.0]. As expected, a significantly lower GDH value was detected in the negative patients (0.26 [0.23–0.29]; *p* < 0.01).

Within the GDH-positive subgroup, 32 out of 60 (53.33%) patients had not received any antibiotics before the diagnosis of CDI. Conversely, 28 out of 60 patients had received antibiotics: 9 patients (15.00%) had received a single class of antibiotics, 6 patients (10.00%) had received two classes, 5 patients (8.33%) had received three classes, 6 patients (10.00%) had received four classes, and 2 patients (3.33%) had received five classes. On the contrary, only three CDI-negative patients had received antibiotic treatment (see Table 1 for details).

As reported in Table 1, a total of nine classes of antibiotics were documented. Antibiotics from the penicillin class were used most frequently; in fact, they had been used in 20 patients, and they had frequently been co-administered with other antibiotics (n = 19). Following penicillin, carbapenem was the second most employed class (n = 11). Trimethoprim/sulfamethoxazole antibiotics had been administered to only two patients, while rifamycin had been prescribed to one patient. In the therapeutic plans implemented before the manifestation of CDI, additional non-CDI-related interventions, such as antifungals or antitubercular agents, had also been considered for specific patients. Unfortunately, no information was available on treatment duration.

### 3.2. Gut Microbiota Analysis

**Figure 2:**
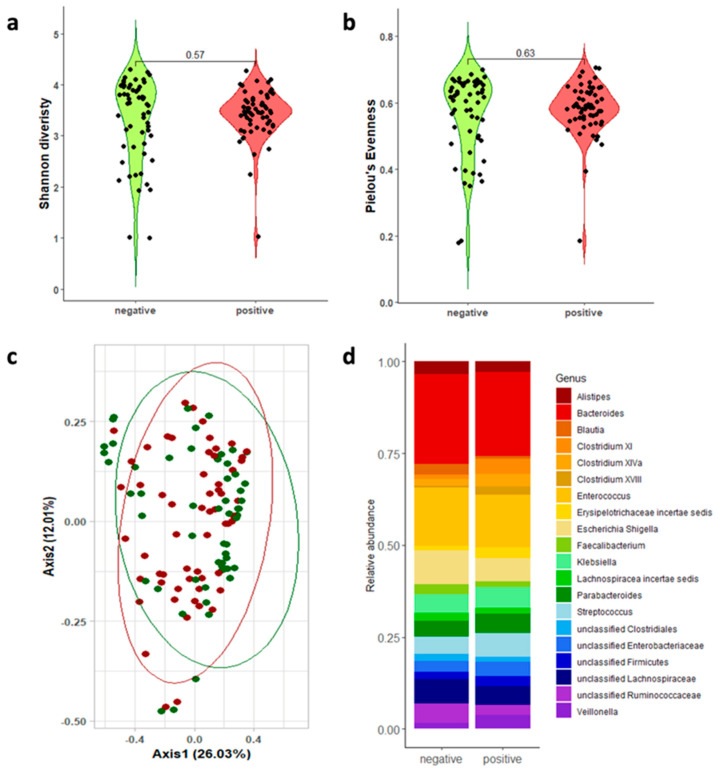
Gut microbiota analysis of the patients with or without CDI by sequencing the V1-V3 (sol.A) 16S RNA hypervariable regions. The alpha diversity was shown as the Shannon diversity index (**a**) and Pielou’s evenness (**b**), with both represented as a combination of violin plots and dot plot charts. The beta diversity was evaluated by using the Bray–Curtis distance and graphically represented using a principal coordinate analysis (PCoA) (**c**). Relative abundance is shown for the top 20 genera as a bar chart (**d**).

**Figure 3:**
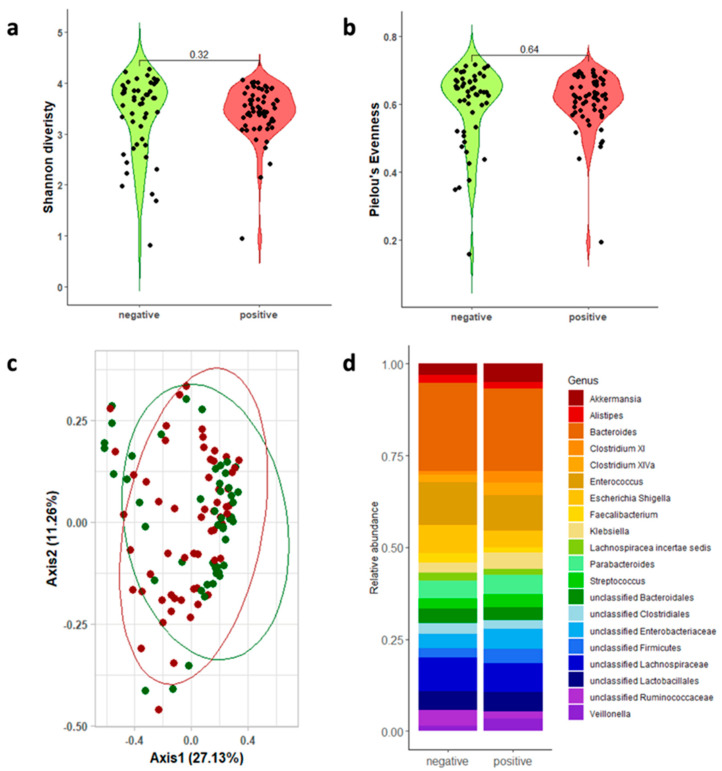
Gut microbiota analysis of the patients with or without CDI by sequencing the V3-V4 and V6 (sol.B) 16S RNA hypervariable regions. The alpha diversity was shown as the Shannon diversity index (**a**) and Pielou’s evenness (**b**), with both represented as a combination of violin plots and dot plot charts. The beta diversity was evaluated by using the Bray–Curtis distance and graphically represented using principal coordinate analysis (PCoA) (**c**). Relative abundance is shown for the top 20 genera as a bar chart (**d**).

We analyzed the gut microbiota of the GDH- and TOX A/B-positive patients compared to that of the GDH-negative patients to identify a gut microbial signature associated with the onset of CDI. Given the absence of global guidelines, we sequenced both the V1-V3 (sol.A) and V3-V4-V6 (sol.B) 16s hypervariable regions. A microbiome analysis was performed on 116 out of 120 samples (96.66%) and 109 out of 120 samples (90.83%) for sol.A and sol.B, respectively, due to the low sample material. A total of 15 specimens (4 for V1-V3 and 11 for V3-V4-V6) did not pass the quality control (a minimum of 40,000 reads). Sample drop-out did not impact the pre-calculated statistical power. A pre-analysis was conducted to remove taxa not seen more than three times in at least 10% of the samples and ultimately obtained median values of 116,180 [87,063–138,244] and 66,501 [49,283–95,161] reads for V1-V3 (Figure 2) and V3-V4-V6 (Figure 3), respectively. No significant differences were shown in the alpha diversity, measured as the Shannon diversity and Pielou’s evenness, between the CDI-positive and CDI-negative patients (Figure 2a,b and Figure 3a,b and Table 2). Similarly, the principal coordinate analysis (PCoA) of the inter-individual variation, based on the Bray–Curtis distance (giving more importance to the community composition rather than how much they differed), indicated no separation between the two groups, as corroborated by the statistical significance in the PERMANOVA (*p* > 0.05; Figure 2c and Figure 3c).

The analysis of both sol.A and sol.B showed six major phyla: *Actinobacteria*, *Bacteroidetes*, *Firmicutes*, *Fusobacteria*, *Proteobacteria*, and *Verrucomicrobia*. As shown in Tables S1 and S2, no statistical differences were detected at the phylum level. Indeed, the slight variation in relative abundance was accounted for at the interanalysis and intergroup (CDI-positive or CDI-negative) levels.

Conversely, some different features were highlighted at the genus level when the sol.A or sol.B 16s regions were sequenced. The top 20 genera overall and the statistical analysis are reported in Tables S3 and S4. As shown in Figure 2d (V1-V3 top 20 genera), *Clostridium* XI (*p* < 0.001), *Clostridium* XIVa (*p* = 0.0015), *Clostridium* XVIII (*p* = 0.0018), *Faecalibacterium* (*p* = 0.034), and *Blautia* (*p* = 0.0035) were significantly different between the CDI-negative and CDI-positive samples. The relative abundance of *Clostridium* XI, *Clostridium* XIVa, and *Clostridium* XVIII increased in the CDI-positive patients compared to that in the CDI-negative patients (3.9%, 3.3%, and 2.4% versus 1.2%, 1.7%, and 0.4%, respectively). On the contrary, *Blautia* and *Faecalibacterium* decreased in the CDI patients, showing average values of 0.9% and 1.6%, respectively. The V3-V4-V6-based analysis identified significant differences in *Clostridium* XI (*p* < 0.001), *Clostridium* XIVa (*p* = 0.0098), *Enterococcus* (*p* = 0.046), *Alistipes* (*p* = 0.049), *Veillonella* (*p* = 0.036), and *Faecalibacterium* (*p* = 0.00015) (Figure 3d).

**Figure 4:**
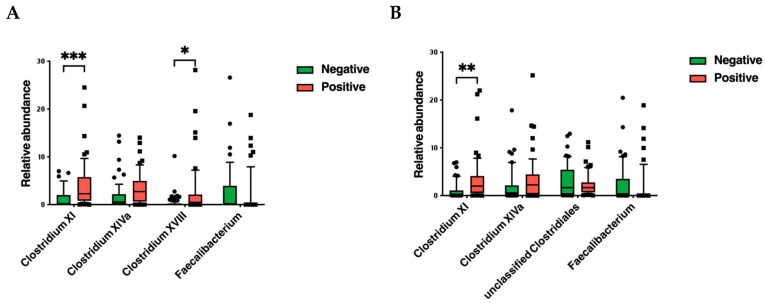
Evaluation of the relative abundance of the genera *Clostridium* XI, *Clostridium* XIVa, *Clostridium* XVIII, and *Faecalibacterium* in CDI-positive and CDI-negative patients using microbiota solution A (**A**) and microbiota solution B (**B**). Data are reported for each sample together with a box plot with organization according to Tukey’s method. (*, *p* < 0.05; **, *p* < 0.01; ***, *p* < 0.001).

*Clostridium* XI, representing the cluster of *C. difficile*, followed an increased trend in both analyses (Figure 4). Intriguingly, the beneficial genera *Clostridium* XIVa and Clostridium XVIII showed an overall increase in the CDI patients (Figure 4). Conversely, the *Faecalibacterium* genus showed an averaged relative abundance that was ten times higher (~4%) in the CDI-negative patients compared to the CDI patients. Even though this was not statistically significant, for the *Akkermansia* genus, revealed only when the V3-V4-V6 16s regions were sequenced, average values of 4.9% were shown in the CDI-positive patients, with respect to the 3.0% measured in the CDI-negative patients.

**Figure 5:**
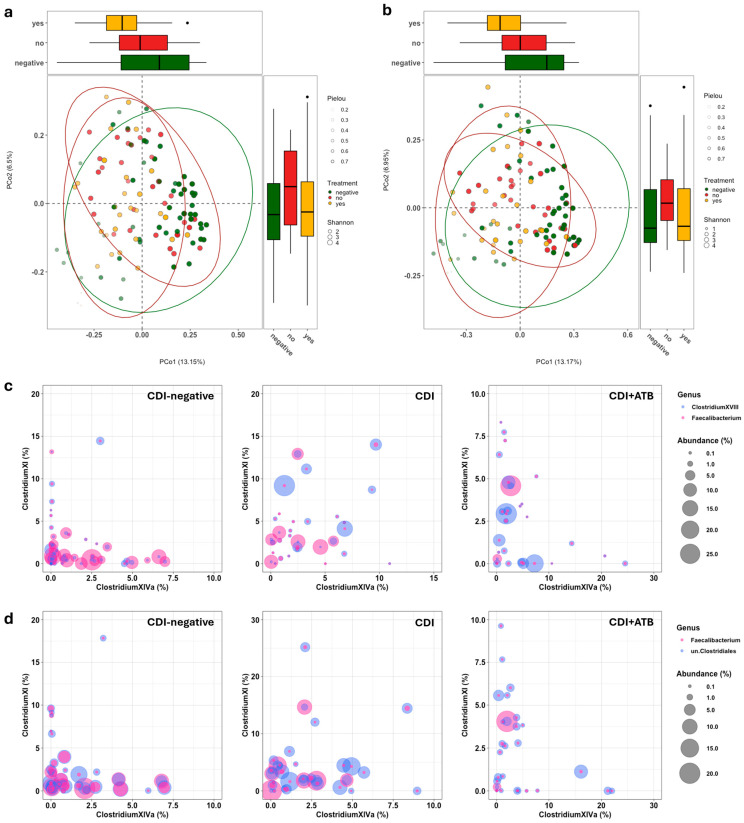
Impact of the antibiotic treatment on the gut microbiota of patients with or without CDI by sequencing the V1-V3 (sol.A, (**a**)) and V3-V4 and V6 (sol.B, (**b**)) 16S RNA hypervariable regions. Beta diversity, evaluated by using the Bray–Curtis distance and graphically represented using a principal coordinate analysis (PCoA), was reported (in green, CDI-negative samples; in red and gold, CDI samples or CDI samples of patients who had been administered the antibiotic treatment, respectively). Size and color opacity are used to depict the Shannon diversity index and Pielou’s evenness. Bubble plot graphs (sol.A and sol.B: (**c**) and (**d**), respectively) report the relative abundance of the genera *Clostridium* XI (y-axis) and *Clostridium* XIVa (x-axis), *Faecalibacterium*, and *Clostridium* XVIII (sol.A) or *unclassified Clostridiales* (sol.B).

To elucidate the impact of the antibiotic treatment, we analyzed samples from the CDI-negative patients, CDI-positive patients, and CDI-positive patients to whom antibiotic treatment was administered (independently of the type of treatment) by performing a beta diversity analysis according to the Bray-Curtis distance (Figure 5). While both regions sequenced (sol.A and sol.B) showed comparable results, slight differences were evidenced among the three groups. The bubble plots indicated a trend of *C. difficile* XI’s (4.69% for sol.A; 4.07% for sol.B) increased relative abundance following the antibiotic treatment and a strong and significant decrease in *Faecalibacterium* (0.81% for sol.A; 0.78 for sol.B) in comparison with both the untreated CDI-positive samples and the CDI-negative samples (Figure 5c,d and Tables S1 and S2). These results suggested no macroscopic differences at the phylum level or in the alpha and beta diversity metrics between CDI-positive and CDI-negative patients. Conversely, at the genus level, slight but significant variations, which prompted the following antibiotic treatment, were detected.

## 4. Discussion

The idea of the existence of a direct link between a disrupted gut microbiome, the use of antibiotics, and the development of CDI has become embedded into the scientific community, so in some countries, the rates of CDI are used to evaluate the efficacy of a hospital’s antibiotic stewardship policies [38]. Nevertheless, numerous cases of community-acquired CDI have been reported in the literature, and even in our cohort, we have reported cases of CDI in antibiotic-naïve patients [37].

CDI is usually suspected in patients with acute diarrhoea, and a diagnosis can be established through several laboratory techniques such as NAATs and enzyme immunoassays (EIAs) for the *C. difficile* GDH antigen and for *C. difficile* toxins A/B, as well as a selective anaerobic culture [5,39].

New tools in CDI diagnosis are necessary in order to contribute not only to treatment efficacy but also the implementation of appropriate isolation techniques and patient safety [40]. 16S rRNA gene sequencing for microbiome profiling may have a role in destigmatizing CDI, which is often considered to be a hospital-acquired infection, and identifying specific risk and protective factors that may also be useful in choosing stool donors for fecal transplants.

We investigated the gut microbiota composition in a cohort of patients at the onset, and first detection, of CDI compared to that of negative individuals. Furthermore, we analyzed fecal samples by sequencing the V1-V3 and V3-V4-V6 16s RNA hypervariable regions using tests that complied with the European In Vitro Diagnostic Devices Directive (CE-IVD).

At the onset of CDI, there were no differences in the alpha diversity metrics between the *C. difficile*-positive patients and the CDI-negative individuals. Indeed, no changes were detected in either the number of species which composed the gut’s microbial community or in the prevalence of certain species. As reported in the beta diversity analysis, the absence of a discernible separation between these two groups was aligned with earlier findings reported in the literature [20,41].

As highlighted in a recent review [42], when CDI occurs, numerous genera belonging to *Clostridium* and *Veillonella* significantly increase. Particularly, the *Clostridium* XI genus, which includes *C. difficile*, was enhanced in the CDI-positive samples, similarly to the *Clostridium* XIVa and *Clostridium* XVIII genera. The increase in genera such as *Clostridium* XI and *Clostridium* XIVa was mainly attributed to the antibiotic therapies undergone by the patients prior to the detection of CDI [43]. While the increased prevalence of *Clostridium* XI in CDI can be explained by the presence of opportunistic species in this cluster in addition to *C. difficile* [43], the enhancement in the clusters *Clostridium* XIVa and *Clostridium* XVIII remains much more elusive. Interestingly, many inflammatory diseases have been linked to a pronounced depletion of the *Clostridiales* organisms belonging to these clusters [20,43,44,45]. *Clostridium* clusters XIVa, IV, and XVIII appear to stimulate the differentiation of Treg cells, so microbial imbalances may disrupt the regulatory processes that suppress inflammation. The oral administration of *Clostridia* belonging to these clusters has been demonstrated to attenuate colitis and allergic diarrhoea, improving gut immune tolerance [45], suggesting their roles as probiotics [45,46]. The slight increase in gut-resident *Clostridium* XIVa and *Clostridium* XVIII may have resulted as a last attempt to counteract the *Clostridium* XI (*C. difficile*)-promoted inflammation. Several studies have described *Faecalibacterium prausnitzii* as having a protective effect against gut inflammation, including in CDI [47,48]. For all of these reasons, we hypnotize that variations in the *Clostridium*/*Faecalibacterium* ratio may represent a microbiota biomarker useful for prompting the investigation of CDI using specific tests.

In line with our hypothesis, in treated CDI-positive patients, *Clostridium* XIVa and *Faecalibacterium* decreasing due to antibiotic treatments may promote a significant impairment in the gut microbiota that indirectly promotes CDI. Importantly, this antibiotic-associated change may persist after the antibiotic therapy has ended for several days, delaying healing and promoting recurrent infections or other diseases [49,50].

The other genera were primarily detected in one of the two analyses. *Akkermansia* and *Erysipelotrichaceae* were exclusively identified in the V3-V4-V6-based analysis, as were unclassified *Bacteroidales* and *Lactobacillales* families. This discrepancy arises from variations in the taxonomic classification across different variable regions of the genetic sequences, representing a challenge in conducting cross-study comparisons and introducing additional biases into compositional analyses [51]. Indeed, short-amplicon primers may not exhibit the desired universality, further complicating taxonomic classification and leading to the misclassification of closely related bacterial species and genera, which may become indistinguishable in the analysis [52,53].

Identification of the genus *Akkermansia* may denote a healthy state, as evidenced by its characterization as a probiotic in several studies related to CDI [54,55,56]. Our results showed the V3-V4-V6 hypervariable region analysis as the most useful tool for assessing the presence of this genus, as well as its variations. These results are comparable with those of other studies that highlighted the use of the V3-V4 regions to detect the *Akkermansia* genus, even though its relative abundance appeared lower than the measures obtained using quantitative PCR [57,58].

Although *Akkermansia muciniphila* has been described to ameliorate the host’s metabolic health and intestinal homeostasis, its function remains still elusive and complex [59]. It has been noted that the *Akkermansia* genus’s relative abundance rapidly decreases when it is included in a bacterial consortium administrated to patients with recurrent CDI [60]. Even though *Akkermansia* is widely considered one of the next-generation probiotics, its abundance needs to be monitored because its overgrowth may be linked to inflammatory diseases [61,62]. In this context, an increased abundance of *Akkermansia* and a decrease in *Faecalibacterium*, together with an enhanced abundance of *Clostridium* XI, may be possible biomarkers related to the development of CDI.

Gut microbiota analysis is booming as an innovative approach to diagnosing several diseases, but more studies are needed to evaluate its potential use as an adjunctive test for CDI diagnosis. In this scenario, 16S rRNA gene profiling may not replace the tests routinely used for CDI diagnosis, but it might be useful in providing a more complete picture and providing an assessment of the *C. difficile* levels within the broader context of microbiota profiling, as well as offering valuable insights for CDI surveillance. This approach may have potential applications in predicting disease outcomes, exploring new therapeutic avenues for CDI, and the detection of *C. difficile* following gut microbiota analysis, along with the evaluation of other clinical signs or the evolution of a patient’s clinical condition. The major limitation of this study may be the restricted number of patients, although stringent categorization of the patients was carried out (for example, the narrow intragroup age range and comparable intergroup differences). Of note, our results appear to be in line with other recent results: in particular, the relative abundance at the phylum level was comparable for the patients defined as colonized by *C. difficile*, suggesting that our observations may support its use as a biomarker for CDI onset [47,63]. On the other hand, microbiota snapshots from cross-sectional studies, including this study, offer limited information regarding the dynamics of microbial communities and thus are not well suited for inferring the causal relationships between *C. difficile* colonization and infection and host components, leading to stochastic findings [64]. Indeed, understanding these complex dynamics remains a challenge nowadays due to the influence of several factors related to the host and the environment which can generally impair the microbiota [65].

## 5. Conclusions

It is fundamental to underline that in profiling the gut microbiota associated with the onset of CDI, it is particularly challenging to determine the extent of the antibiotic treatments that patients have undergone prior to the the detection of this pathogen [4].

Nevertheless, accurate identification of *C. difficile* and related taxa remains pertinent, especially in the evaluation of the clinical impact of emerging microbial therapies. The utilization of 16S rRNA gene sequencing to validate the recovery of the microbiota may contribute to an advancement in our understanding of the microbial shifts associated with CDI, as presented in this study.

Although the ratio of *Clostridium*/*Faecalibacterium* bacteria offers a potential strategy for improving the diagnosis of CDI, further investigation in larger patient cohorts is required to validate these findings and examine the efficacy of targeted microbiota-based therapies for the prevention and treatment of CDI. Longitudinal studies, appropriate models, and shared standardized methods will play a key role in elucidating the role of the gut microbiota in the development of CDI, its recurrence, and recovery from causal interactions.

## Figures and Tables

**Table 1:** Features of 120 patients investigated for *Clostridioides difficile* by using Chemiluminescence Immunoassay searching for Glutamate Dehydrogenase and toxins A/B. Data were reported as mean, median, and 25th–75th percentile or as total number (percentage). * “Other” refers to the following classes of drugs: antituberculars, azoles, echinocandins, fosfomycin, oxazolidinones.

Parameter	CDI-Positive (n. 60)		CDI-Negative (n. 60)
Gender, n (%) Female	26 (43.3)		33 (55.0)
Age (years, median [Q1–Q3])	75.50; [62.00–82.00]		62 [48.50–76.00]
Hospitalization (days, median [Q1–Q3])	9.50; [2.00–24.75]		4.50 [1.00–10.75]
Community-acquired infections, n (%)	23 (38.3)		-
Dead patients, n (%)	5 (74.0)		5 (74.0)
GDH (median [Q1–Q3])	95.00; [95.00–95.00]		0.26 [0.23–0.29]
Toxins A/B (median [Q1–Q3])	10.1; [3.17–71.00]		-
Number of antibiotics and antibiotic classes received before onset of CDI, n (%)			
0	32 (53.33)		57 (95.00)
1 Carbapenem (1); Cephalosporins (2); Glycopeptide (1); Penicillins (5).	9 (15.00)	1 Rifamycin (1); Quinolones (1); Other (1). *	3 (5.00)
2 Carbapenems (2); Cephalosporin (1); Macrolide (1); Penicillins (5); Trim/Sulfa (1); Other * (1).	6 (10.00)		-
3 β-Lactam (1); Carbapenems (3); Cephalosporin (1); Penicillins (3); Other * (3).	5 (8.33)		-
4 β-Lactam (1); Carbapenems (3); Cephalosporin (1); Glycopeptide (1); Macrolides (3); Penicillins (6); Quinolones (3); Rifamycin (1); Trim/Sulfa (1); Other * (5).	6 (10.00)		-
5 Carbapenems (2); Cephalosporin (1); Glycopeptides (3); Penicillin (1); Other * (3).	2 (3.33)		-

**Table 2:** The alpha diversity index detected using microbiota solution A (V1-V3) and solution B (V3-V4 and V6) in *Clostridioides difficile* infection (CDI)-positive patients and CDI-negative controls. Data were reported as the mean, median and 25th–75th percentile. Wilcoxon’s statistical test for unpaired data was used to assess the statistical significance.

	16s rRNA Regions	CDI-Positive Mean; Median [Q1–Q3]	CDI-Negative Mean; Median [Q1–Q3]	*Wilcoxon*
Shannon diversity	V1-V3	3.424; 3.468 [3.221–3.697];	3.330; 3.637 [2.844–3.955]	*p* = 0.565
	V3-V4-V6	3.432; 3.495 [3.227–3.778]	3.391; 3.699 [2.964–3.946]	*p* = 0.317
Pielou’s evenness	V1-V3	0.581; 0.586 [0.550–0.632];	0.564; 0.617 [0.500–0.656]	*p* = 0.626
	V3-V4-V6	0.609; 0.622 [0.580–0.633]	0.596; 0.636 [0.543–0.670]	*p* = 0.637

## Data Availability

The raw sequence data generated during the current study are available in the Sequence Read Archive (SRA) under the following BioProject accession number: PRJNA1106051. The original contributions presented in this study are included in the article; further inquiries and information on the analysis can be directed to the corresponding author.

## Supplementary Materials

The following supporting information can be downloaded at https://www.mdpi.com/article/10.3390/microorganisms13010016/s1. Table S1: Relative abundance at the phylum level ascertained using microbiota solutions A and B in CDI-positive patients and CDI-negative controls; Table S2: Statistical analysis conducted on relative abundance at the phylum level to investigate the differences between microbiota solution A and microbiota solution B in CDI-positive and CDI-negative patients. Table S3: Relative abundance ascertained at the genus level with microbiota solutions A and B in CDI-positive patients and CDI-negative controls. Data are reported as the mean, median, and 25th-75th percentile. Table S4: Statistical analysis conducted on relative abundance at the genus level to investigate the differences between microbiota solution A and microbiota solution B in CDI-positive and CDI-negative patients. Table S5: Relative abundance of 5 genera among 3 groups (antibiotic treatment, no treatment, and negative) obtained following sequencing with microbiota solution A and microbiota solution B. Table S6: Wilcoxon’s signed rank test of the bacterial genera among the 3 different groups (treated CDI, non-treated CDI, and CDI-negative) for microbiota solution A and microbiota solution B.
