# Supplementary material for: Can Gut Microbiota Analysis Reveal Clostridioides difficile Infection? Evidence from an Italian Cohort at Disease Onset
Source: Microorganisms. 2024 Dec 25;13(1):16. doi: 10.3390/microorganisms13010016 (PMC11767363; doi:10.3390/microorganisms13010016)
Supplement: Supplementary file 1 [file microorganisms-13-00016-s001.zip › microorganisms-3386228-supplementary.pdf]

Supplementary table S1. Phylum relative abundances obtained with Microbiota solution A and solution B in CDI positive patients and CDI negative controls. Data were reported as mean, median and 25th-75th percentile.

	**Microbiota Solution A**		**Microbiota Solution B**	
**Phylum**	**CDI-negative Mean; median [Q1-Q3]**	**CDI-positive Mean; median [Q1-Q3]**	**CDI-negative Mean; median [Q1-Q3]**	**CDI-positive Mean; median [Q1-Q3]**
Actinobacteria	0,01343; 0,00383 [0,00075-0,01245]	0,01417; 0,00809 [0,00161-0,02513]	0,0372; 0,0164 [0,0045-0,0433]	0,02493; 0,01678 [0,00348-0,02988]
Bacteroidetes	0,29928; 0,32580 [0,05459-0,43700]	0,27266; 0,31735 [0,09575-0,42262]	0,3130; 0,3379 [0,0804-0,4604]	0,28294; 0,35761 [0,07518-0,42761]
Firmicutes	0,51371; 0,50400 [0,35867-0,62879]	0,52062; 0,53499 [0,41350-0,60583]	0,4850; 0,4807 [0,3233-0,6184]	0,48370; 0,44510 [0,38041-0,55925]
Fusobacteria	0,00557; 0,00013 [0,00009-0,00023]	0,02468; 0,00015 [0,00009-0,00141]	0,0035; 0,0001 [0,0000-0,0002]	0,01893; 0,00006 [0,00001; 0,00171]
Proteobacteria	0,16782; 0,07945 [0,02377-0,22776]	0,16746; 0,15285 [0,04019-0,26667]	0,1380; 0,0594 [0,0183-0,2088]	0,15101; 0,08688 [0,03271-0,22986]
Verrucomicrobia	0,00020; 0,00001 [0,00000-0,00007]	0,00041; 0,00002 [0,00000-0,00009]	0,0234; 0,0002 [0,0001-0,0027]	0,03849; 0,00021 [0,00006-0,00350]

Supplementary table S2. Statistical analysis conducted on phylum relative abundances to investigate the differences between Microbiota solution A and Microbiota solution B in CDI-positive and CDI-negative patients. Wilcoxon statistical test for unpaired data was used to assess statistical significance. Data were represented as *p* value (*p*).

	**Microbiota solution A**	**Microbiota solution B**
Phylum	**Wilcoxon *p* value**	
Actinobacteria	*0.517*	*0.102*
Bacteroidetes	*0.537*	*0.528*
Firmicutes	*0.791*	*0.630*
Fusobacteria	*0.467*	*0.222*
Proteobacteria	*0.261*	*0.328*
Verrucomicrobia	*0.765*	*0.396*

Supplementary table S3. Genus relative abundances obtained with Microbiota solution A and solution B in CDI positive patients and CDI negative controls. Data were reported as mean, median and 25th-75th percentile.

	Microbiota Solution A		Microbiota Solution B	
**Genus**	**CDI-negative Mean; median [Q1-Q3]**	**CDI-positive Mean; median [Q1-Q3]**	**CDI-negative Mean; median [Q1-Q3]**	**CDI-positive Mean; median [Q1-Q3]**
Akkermansia	NA	NA	0,03010; 0,00024 [0,00010-0,00386]	0,04928; 0,00027 [0,00009-0,00423]
Alistipes	0,0337; 0,0121; [0,0003-0,0489]	0,0279; 0,0007 [0,0003-0,0373]	0,02262; 0,00936 [0,00022-0,03976]	0,01895; 0,00038 [0,00009-0,01783]
Bacteroides	0,2455; 0,2621 [0,0255-0, 3773]	0,2291; 0,2149 [0,0211-0,3699]	0,23916; 0,26170 [0,03442-0,35265]	0,22454; 0,23161 [0,01119-0,37714]
Blautia	0,0297; 0,0074 [0,0004-0,0421]	0,0092; 0,0013 [0,0001-0,0076]	NA	NA
***Clostridium* XI**	0,0117; 0,0011 [0,0003-0,0187]	0,0395; 0,0228 [0,0083-0,0544]	0,01027; 0,00070 [0,00008-0,00887]	0,03178; 0,01986 [0,00614-0,04076]
***Clostridium* XlVa**	0,0178; 0,0057 [0,0004-0,0210]	0,0332; 0,0275 [0,0065-0,0490]	0,01908 0,00569 [0,00126-0,02097]	0,03378; 0,02240 [0,00321-0,04371]
***Clostridium* XVIII**	0,0049; 0,0009 [0,0002-0,0023]	0,0240; 0,0050 [0,0003-0,0195]	NA	NA
Enterococcus	0,1581; 0,0146 [0,0021-0,2096]	0,1432; 0,0683 [0,0153-0,2102]	0,11831; 0,00317 [0,00051-0,11515]	0,09630; 0,02777 [0,00659-0,11066]
***Erysipelotrichaceae* incertae sedis**	0,0133; 0,020 [0,0003-0,0072]	0,0299; 0,0105 [0,0007-0,0333]	NA	NA
Escherichia Shigella	0,0906; 0,0102 [0,0008-0,0964]	0,0615; 0,0059 [0,0004-0,0505]	0,07711; 0,00186 [0,00033-0,04802]	0,04482; 0,00331 [0,00031-0,02973]
Faecalibacterium	0,0270; 0,0015 [0,0003-0,0380]	0,0160; 0,0004 [0,0003-0,0011]	0,02447; 0,00365 [0,00038-0,03261]	0,01447; 0,00033 [0,00019-0,00104]
Klebsiella	0,0519; 0,0005 [0,0003-0,0025]	0,0569; 0,0004 [0,0002-0,0692]	0,02755; 0,00009 [0,00001-0,00074]	0,04495; 0,00019 [0,00004-0,02409]
***Lachnospiracea* incertae sedis**	0,0206; 0,0038 [0,0004-0,0305]	0,0159; 0,0031 [0,0004-0,0172]	0,02067; 0,00831 [0,00094-0,03411]	0,01622; 0,00370 [0,0003-0,01569]
Parabacteroides	0,0437; 0,0191 [0,0008-0,0430]	0,0532; 0,0109 [0,0005-0,0925]	0,05039; 0,01871 [0,00071-0,04424]	0,05317; 0,00209 [0,00032-0,09259]
Streptococcus	0,0469; 0,0146 [0,0033-0,0747]	0,0639; 0,0153 [0,0027-0,0617]	0,02655; 0,00406 [0,00084-0,03776]	0,03522; 0,00487 [0,00073-0,01990]
unclassified Bacteroidales	NA	NA	0,03933; 0,04189 [0,00737-0,05465]	0,03512; 0,02787 [0,00375-0,06178]
**unclassified *Clostridiales***	0,0206; 0,0086 [0,0010-0,0388]	0,0141; 0,0097 [0,0035-0,0170]	0,03011; 0,01665 [0,00217-0,04962]	0,02193; 0,01668 [0,00726-0,02652]
**unclassified *Enterobacteriaceae***	0,0292; 0,0118 [0,0012-0,0407]	0,0397; 0,0181 [0,0030-0,0483]	0,03813; 0,01994 [0,00072-0,05025]	0,05476; 0,02379 [0,00321-0,06164]
**unclassified *Firmicutes***	0,0191; 0,0132 [0,0031-0,0303]	0,0258; 0,0211 [0,0129-0,0356]	0,02654; 0,01600 [0,00492-0,03766]	0,03927; 0,02776 [0,01844-0,05139]
**unclassified *Lachnospiraceae***	0,0659; 0,0556 [0,0044-0,1035]	0,0511; 0,0411 [0,0170-0,0701]	0,09139; 0,07769 [0,01750-0,14584]	0,07878; 0,06116 [0,02035-0,10404]
**unclassified *Lactobacillales***	NA	NA	0,05081; 0,02409 [0,00251-0,06711]	0,05256; 0,03705 [0,01790-0,07586]
**unclassified *Ruminococcaceae***	0,0527; 0,0274 [0,0011-0,0846]	0,0284; 0,0038 [0,0010-0,0385]	0,04374; 0,01925 [0,00226-0,06830]	0,01989; 0,00498 [0,00089-0,03037]
Veillonella	0,0170; 0,0003 [0,0002-0,0026]	0,0376; 0,0015 [0,0002-0,0428]	0,01369; 0,00019 [0,00003-0,00163	0,03422; 0,00095 [0,00007-0,02938]

Supplementary Table S4. Statistical analysis conducted on genus relative abundances to investigate the differences between Microbiota solution A and Microbiota solution B in CDI-positive and CDI-negative patients. Wilcoxon statistical test for unpaired data was used to assess statistical significance. Data were represented as *p* value (*p*).

	**Microbiota solution A**	**Microbiota solution B**
**Genus**	**Wilcoxon *p* value**	
Akkermansia	NA	0,8409
Alistipes	0,2223	0,0491
Bacteroides	0,6270	0,6074
Blautia	0,0035	NA
Clostridium XI	0,0000	0,0000
Clostridium XlVa	0,0015	0,0098
Enterococcus	*0,2223*	0,0464
Clostridium XVIII	*0,0018*	NA
Erysipelotrichaceae incertae sedis	0,0053	NA
Escherichia Shigella	0,2223	0,5055
Faecalibacterium	0,0340	0,0001
Klebsiella	0,8467	0,0937
Lachnospiracea incertae sedis	0,3593	0,1780
Parabacteroides	0,8771	0,9152
Streptococcus	0,9868	0,8624
unclassified Bacteroidales	NA	0,5905
unclassified Clostridiales	*0,8252*	0,9733
unclassified Enterobacteriaceae	*0,3478*	0,3929
unclassified Firmicutes	*0,0163*	0,0066
unclassified Lachnospiraceae	*0,2941*	0,3665
unclassified Lactobacillales	NA	0,0451
unclassified Ruminococcaceae	*0,0900*	0,0250
Veillonella	*0,0859*	0,0360

Supplementary Table S5. Relative abundances of 5 genera among 3 groups (antibiotic treatment, no treatment and negative) obtained following sequencing with Microbiota solution A and Microbiota solution B.

**Treatment**	**Genus**	**Solution A**<br>**Mean; median [Q1-Q3]**	**Solution B**<br>**Mean; median [Q1-Q3]**
CDI+ATB			
	Clostridium XI	0,0469; 0,0220 [0,0105-0,0530]	0,0407; 0,0182 [0,0081-0,0407]
	Clostridium XlVa	0,0252; 0,0195 [0,0007-0,0378]	0,0244; 0,0169 [0,0002-0,0403]
	Clostridium XVIII	0,0234; 0,0072 [0,0003-0,0188]	NA
	Unclassified *Clostridiales*	NA	0,0135; 0,0096 [0,0041-0,0172]
	Faecalibacterium	0,0081; 0,0004 [0,0003-0,0005]	0,0078; 0,0003 [0,0002-0,0004]
CDI			
	Clostridium XI	0,0327; 0,0243 [0,0080-0,0539]	0,0237; 0,0199 [0,0049-0,0355]
	Clostridium XlVa	0,0405; 0,0284 [0,0149-0,0512]	0,0422; 0,0224 [0,0116-0,0449]
	Clostridium XVIII	0,0245; 0,0028 [0,0004-0,0192]	NA
	Unclassified *Clostridiales*	NA	0,0295; 0,0235 [0,0101-0,0371]
	Faecalibacterium	0,0232; 0,0004 [0,0003-0,0068]	0,0205; 0,0004 [0,0003-0,0043]
CDI-Negative			
	Clostridium XI	0,0117; 0,0011 [0,0003-0,0187]	0,0103; 0,0007 [0,0001-0,0089]
	Clostridium XlVa	0,0178; 0,0057 [0,0004-0,0210]	0,0191; 0,0057 [0,0013-0,0210]
	Clostridium XVIII	0,0049; 0,0009 [0,0002-0,0023]	NA
	Unclassified *Clostridiales*	NA	0,0301; 0,0167 [0,0022-0,0496]
	Faecalibacterium	0,0270; 0,0015 [0,0003-0,0380]	0,0245; 0,0036 [0,0004-0,0326]

Supplementary Table S6. Wilcoxon Signed rank test of bacterial genera among 3 different groups (Treated CDI, Not Treated CDI, and CDI Negative) for Microbiota Solution A and Microbiota Solution B.

Genus	CDI+ATB vs CDI<br>*p* value	CDI+ATB vs CDI-Negative<br>*p* value	CDI vs CDI-Negative<br>*p* value
**Solution A**			
Clostridium XI	0,735	< 0,001	0,0001
Clostridium XVIII	0,792	0,008	0,014
Clostridium XlVa	0,097	0,174	< 0,001
Faecalibacterium	0,294	0,010	0,341
**Solution B**			
Clostridium XI	0,582	< 0,001	0,0001
Clostridium XIVa	0,206	0,229	0,0027
Uncalssified Clostridiales	0,002	0,250	0,2576
Faecalibacterium	0,009	< 0,001	0,0778
